# From efficacy to effectiveness of pharmacological and psychosocial interventions

**DOI:** 10.1192/j.eurpsy.2025.158

**Published:** 2025-08-26

**Authors:** A. Fiorillo

**Affiliations:** University of Campania “Luigi Vanvitelli”, Naples, Italy

## Abstract

**Abstract:**

European citizens suffering from mental disorders still experience several obstacles in achieving functional recovery. Integration of pharmacotherapy and psychosocial interventions represents an optimal strategy in mental health care, but implementation of psychosocial interventions is rarely available in many European countries. Data from efficacy findings in pharmacological and psychosocial trials to effectiveness in real-world studies will be provided, alongside with a focus on the main barriers and obstacles for implementing psychosocial interventions in clinical practice.

**Disclosure of Interest:**

None Declared

